# Numerical Modeling of Fluid Flow in Solid Tumors

**DOI:** 10.1371/journal.pone.0020344

**Published:** 2011-06-06

**Authors:** M. Soltani, P. Chen

**Affiliations:** Department of Chemical Engineering, University of Waterloo, Waterloo, Ontario, Canada; Rutgers University, United States of America

## Abstract

A mathematical model of interstitial fluid flow is developed, based on the application of the governing equations for fluid flow, i.e., the conservation laws for mass and momentum, to physiological systems containing solid tumors. The discretized form of the governing equations, with appropriate boundary conditions, is developed for a predefined tumor geometry. The interstitial fluid pressure and velocity are calculated using a numerical method, element based finite volume. Simulations of interstitial fluid transport in a homogeneous solid tumor demonstrate that, in a uniformly perfused tumor, i.e., one with no necrotic region, because of the interstitial pressure distribution, the distribution of drug particles is non-uniform. Pressure distribution for different values of necrotic radii is examined and two new parameters, the critical tumor radius and critical necrotic radius, are defined. Simulation results show that: 1) tumor radii have a critical size. Below this size, the maximum interstitial fluid pressure is less than what is generally considered to be effective pressure (a parameter determined by vascular pressure, plasma osmotic pressure, and interstitial osmotic pressure). Above this size, the maximum interstitial fluid pressure is equal to effective pressure. As a consequence, drugs transport to the center of smaller tumors is much easier than transport to the center of a tumor whose radius is greater than the critical tumor radius; 2) there is a critical necrotic radius, below which the interstitial fluid pressure at the tumor center is at its maximum value. If the tumor radius is greater than the critical tumor radius, this maximum pressure is equal to effective pressure. Above this critical necrotic radius, the interstitial fluid pressure at the tumor center is below effective pressure. In specific ranges of these critical sizes, drug amount and therefore therapeutic effects are higher because the opposing force, interstitial fluid pressure, is low in these ranges.

## Introduction

Cancer is the second leading cause of death, causing one of every four deaths in North America [1]. Although the most important treatment is surgical removal of the tumor, the key to a successful cure is often an efficient delivery of anticancer drugs after the surgery. Many new drugs have been developed to eradicate cancer but are ineffective when used in humans for lack of efficient delivery. Moreover, all drugs have possible side effects, such as toxicity to normal cells and the development of drug resistance [2]. Residual tumor cells and re-growth of tumors are common sequels to the use of most of these drugs. The drugs' most noticeable limitation is their inability to reach a targeted area without affecting healthy tissues or cells. The two considerations in effective cancer treatment, from an engineering point of view, are drug transport and drug conversion or reaction at the tumor site [3], [4]. Many drugs cannot be delivered to their targets because of transport limitations. Other drugs induce biochemical reactions in the body that produce toxicity.

It is known that more than 85% of human cancers involve solid tumors, and current chemotherapy depends on the adequate delivery of therapeutic agents to tumor sites [1]. It is also well recognized that the blood supply to a solid tumor is highly heterogeneous [5], [6]. In fact, drug concentration is highest closest to vasculature, well-perfused areas, and on the peripheral walls of the tumor, but very little or no drug reaches 90% of the tumor [7], [8]. However, for successful cancer treatment, all areas of the tumor must be exposed to chemotherapy agents. If just the tumor's outer cells are killed, the tumor will eventually regrow [9].

**Figure 1:**
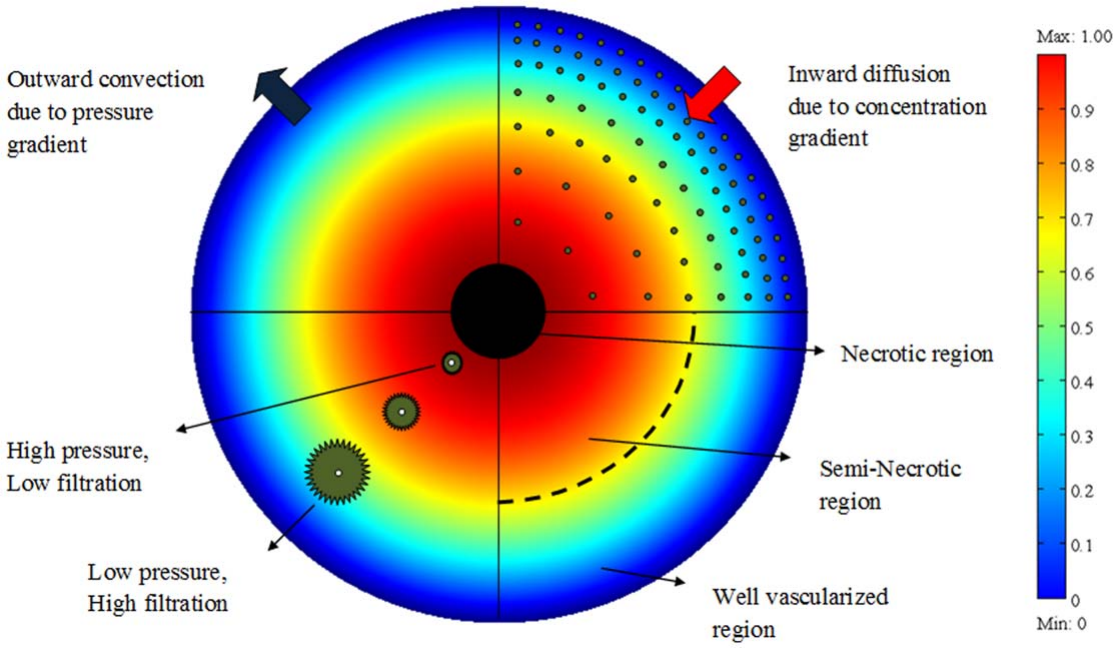
Cross sectional schematic of a solid tumor that shows the three different regions of a solid tumor, IFP distribution, drug concentration and filtration distribution from blood vessels.

According to clinical research findings, even though drug delivery through systemic administration may inhibit tumor growth, most drug treatments fail to eliminate malignant tumors completely [10]. Some experimental and computational investigations show that systemic administration cannot distribute drugs uniformly in tumors. Baxter et al. have shown that, in addition to blood flow heterogeneities and impeded interstitial transport, another mechanism effectively contributes to the non-uniform distribution of drugs: high interstitial pressure in solid tumors [11]–[13]. There are two important effects of this high interstitial pressure, and these effects limit transport in solid tumors. These two effects are illustrated schematically in Fig. 1. The first effect is a decrease in driving force for transcapillary exchange of fluid and therefore the drug. This effect is highlighted in Fig. 1. Low filtration occurs at the center of the tumor as a result of the high interstitial pressure and high filtration occurs at the periphery of the tumor as a result of the low interstitial pressure. The second effect is a radially outward convective flux in the interstitium as fluid flows towards the outer layers of the tumor. This effect is illustrated in Fig. 1 as an outward convection due to pressure gradient. The value of the radially outward fluid velocity at the tumor rim for a tumor with a 1 cm radius, 4.2 g , is 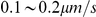 [3]. Another important process in drug delivery is indicated in this schematic, Fig. 1, as an inward diffusion due to concentration gradient of the drug. Effective penetration into a solid tumor requires that the velocity of the diffusion process be higher than that of the convection process [14]. On the other hand, uniformly distributed high interstitial pressure in the center of a tumor blocks convection and, consequently, causes the heterogeneous perfusion of blood into the center of tumors, resulting in the heterogeneous distribution of the drug [13]. The existence of pressure gradients in tumors discovered by Baxter et al. is confirmed by Boucher et al. [15]. Baxter and Jain, using their theoretical framework, further found that the drug diffusivity, pressure and velocity of interstitial fluids, vascular permeability and lymphatic drainage are important factors in determining the drug concentration in tumors [3], [16], [17]. Netti et al. showed that interstitial fluid pressure (IFP) depends on microvascular pressure and blood flow within tumors [18].

Extending the one-dimensional models of Baxter and Jain [3], [16], [17], and Saltzman and Radomsky [19], to a three-dimensional geometry, Wang et al. [20]–[25] developed a simulation framework of drug delivery to tumors. They considered high interstitial pressure in tumors [13], the consequences of blood and lymphatic drainage, and the chemical reaction of the drug with the tissue. The main focus of their work was on using diffusion-convection kinetics to improve simulation result accuracy. They showed that in vitro release profile of the drug from controlled release devices can be combined with state of the art computational fluid dynamics (CFD) simulations to predict the drug delivery behavior, both temporally and spatially, in both normal and cancerous tissues. The focus of their work was the mathematical modeling of the drug release from polymer implants that have certain characteristics of release profile. By applying sensitivity analysis, Zhao et al. [26] determined the effect of spatially changing tissue transport properties on interstitial fluid (including drug particles) transport.

Knowledge of tumor modeling has recently been expanded to include spatial and temporal changes in blood flow by considering capillary network or single vessel approaches [27], [28]. Before Baxter et al. [3], [16], [17] introduced their innovative model of interstitial pressure as a function of tumor radius, little was known about tumor modeling, except that interstitial pressure was highest at the center of a tumor [29] and that pressure is directly proportional to tumor size [29], [30]. The promising combined therapies such as radiotherapy and antiangiogenic therapy in addition to chemotherapy in tumor treatment are good examples that can show the crucial role of the modeling. For example, the computational fluid dynamics modeling results can be applied to optimize the interaction effect of the irradiation to the drug delivery efficiency. The main focus of future drug delivery modeling would be on the transport of the drug in tissues after drug release from either the systemic administration or implantation mechanisms. Modeling in drug delivery involves different processes such as drug diffusion, convective transport in extracellular matrices, drug extravasation from blood vessels, tissue elimination by lymphatic system, and intracellular internalization. In all of these processes, CFD can play a crucial role. To clarify the mechanisms of drug delivery from the injection site to absorption by a solid tumor, computational fluid dynamics has shown promise. So far, drug delivery problems have been most extensively studied with spherical tumors, the simplest to examine with analytical methods. With our proposed numerical method, however, more complex shapes of tumor can be studied. Numerical simulations provide a detailed understanding of the mechanisms of interstitial fluid transport and are also instructive to show some of the major barriers to drug delivery to solid tumors. With this knowledge, one can find, for example, an optimum schedule of drug treatment based on the simulation results. To design an optimum scheme for drug delivery, the transport mechanisms and their obstacles have to be clarified, which is one of the main objectives of this paper.

The proposed CFD model is made for a spherical tumor and its surrounding normal tissue. However, this model can be extended to study non-spherical tumors, especially those geometries reconstructed from high resolution images. The grid generation divides the whole domain or geometry to finite volumes, called meshes. Tetrahedral elements can be used to handle non-spherical tumors. The tumor and its surrounding tissue are assumed to be rigid porous media. The vasculature as a source term varies spatially. Interstitial fluid flow equations in porous media are solved using a CFD code which employs unstructured grids. In studying interstitial fluid pressure distribution, the numerical method, which introduces two critical parameters (tumor radius and necrotic radius), is more effective than the analytical method.

## Methods

### Drug Transport within Solid Tumor

Fluid seeps slowly but constantly from blood vessels into surrounding tissues in most normal tissues. The lymphatic system then reabsorbs this lost fluid and returns it to the blood stream. However, no such lymphatic drainage system for solid tumors has been reported in the literature [3], [4]. Computer simulations show that this lack of lymphatic system involvement may result in a buildup of interstitial pressure, leading to cessation of the usual blood seepage from vessels. As a consequence, large molecules (including cancer-fighting drugs) cannot be carried out of vessels to interact with tissue. Thus, cancer drugs cannot reach the tumor site.

Some drug particles, such as Monoclonal Antibodies (MAbs), are relatively large and move very slowly within tissues [3]. To be effective, these large anticancer agents have to cross the blood vessel wall, traverse the interstitial space that contains the cancer cells, bind to the cancer cell membranes and, if the target is intracellular, penetrate the cancer cell membranes.

Tissue spaces are made up of three parts, all of which are relevant to the delivery of drugs to tumors: the vascular, the interstitial, and the cellular. The vascular space comprises the blood vessels, arteries, arterioles, capillaries, venules, and veins [31]. The interstitial space, a gel-like region between blood vessels and cells, is filled with fibers such as collagen, which gives structural stability, glycosaminoglycans (GAG), and other proteins. The cellular space includes specific tissue cells (cancer cells in a solid tumor), in addition to other cells, such as pericytes, macrophages, and fibroblasts [3], [16], [17].

### Mathematical Model of Interstitial Fluid Transport

The distribution of vasculature and cells in solid tumors is spatially heterogeneous. In the center of solid tumors, there is a necrotic core where most of the cells are dead. The outer boundary of solid tumors contains many exchange vessels, a large blood supply, and fast-dividing cells. Therefore, the mathematical model should be accurate enough to include the dependency of physiological parameters, such as the hydraulic conductivity, on space, that is, it must be able to clearly represent all the physical variations in a tumor. Nevertheless, because the time scale of transport phenomena is much less than that of tumor growth, the physiological parameters can be considered time independent [3]. For the sake of simplicity, solid tumors are considered here to be spherical. In a macroscopic model, only the distribution of variables, such as interstitial pressure and concentration, over the length scale of the tumor radius is important, and microscopic characteristics, such as blood vessels, cells, and the interstitial matrix, are not involved directly in the model. Comparison of the tumor radius, on the order of magnitude of 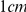, 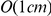, with the intercapillary distance (the average distance between two capillaries), 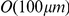, indicates that variations over microscopic length scales can be averaged out [32]. The screening length, 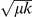 (in which 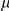 and 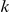 are the viscosity of the interstitial fluid and the hydraulic conductivity of the interstitium, respectively), is on the order of Å; therefore, the fluid transport in the tumor interstitium can be described by Darcy's law for flow through a porous medium [4], [32]–[36]:
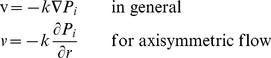
(1)where 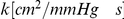, 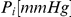, 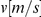 and 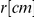 are the hydraulic conductivity of the interstitium, the interstitial fluid pressure, the interstitial fluid velocity and the radial position, respectively. In the case of anisotropic and heterogeneous porous media, 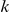 is a tensor and function of the location in the medium. There are some limitations to the use of Darcy's law. For instance, it is not applicable for non-Newtonian fluids, Newtonian fluids at high velocities, or for gases at very low or very high velocities. It is also shown that the friction within the fluid and exchange of momentum between the fluid and solid phases is neglected by Darcy's law. Fortunately, in the interstitium of biological tissues, all these exceptional cases are rare (most of the phenomena are low velocity for Newtonian fluids) except for the friction within the fluid; therefore, Darcy's law is quite applicable to the analysis of interstitial fluid flow.

The mass balance equation for a steady state incompressible fluid is that the divergence of the fluid is zero, or mathematically,
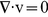
(2)


**Figure 2:**
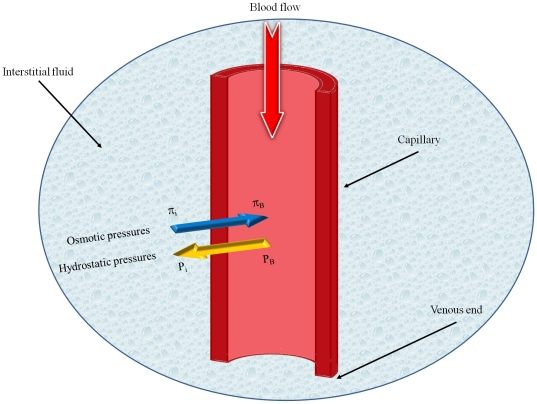
Capillary microcirculation schematic and different types of pressure.

The same equation is also applicable in porous media if there is no fluid source or fluid sink in the medium. However, in most biological tissues, sources and sinks are present. For instance, between interstitial space and the blood or lymph vessels, fluid is exchanged; therefore, the steady state incompressible form of the continuity equation must be modified as
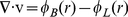
(3)where 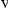 is the fluid velocity in the representative elementary volume (REV). The continuity equation can also be written as
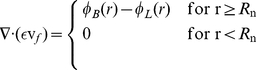
(4)where 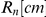, 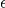, 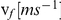, 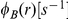, and 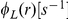 are the radius of the necrotic core, the porosity or the volume fraction of fluid, the fluid velocity averaged in the volume of fluid phase, the fluid source term, and the lymphatic drainage term, respectively. In biological tissues, the two last terms signify the rate of fluid flow per unit volume from blood vessels into the interstitial space and from the interstitial space into lymph vessels, respectively. Both rates can be evaluated through Starling's law. It should be noted that Eq. (4) in this general form is applicable to any kind of biological tissue, whether normal or cancerous. In dead tissues, with no flow in the blood or lymph vessels, the value for both terms is zero. The fluid source term is governed by Starling's law as follows [37], [38]:
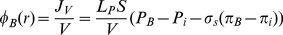
(5)The parameters used in Eq. (5) are: 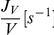, volumetric flow rate out of the vasculature per unit volume of tissue; 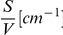, surface area per unit volume for transport in the tumor; 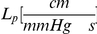, hydraulic conductivity of the microvascular wall; 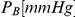, vascular pressure; 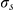, average osmotic reflection coefficient for plasma proteins; 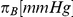, osmotic pressure of the plasma; and 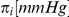, osmotic pressure of the interstitial fluid. Different types of pressure used in Eq. (5) are shown in Fig. 2. It should be noted that the lymphatic drainage term is proportional to the pressure difference between the interstitium and the lymphatics:
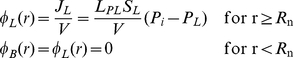
(6)The parameters used in these equations are: 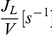, volumetric flow rate into the lymphatics; 
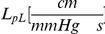
, hydraulic conductivity of the lymphatic wall; and 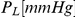, hydrostatic pressure of the lymphatics.

Combination of Darcy's law and the continuity equation results in
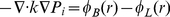
(7)


For a very special case, when 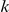 is constant and there are no sinks and sources, the interstitial pressure can be expressed by the very well-known Laplace equation.
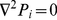
(8)


If all parameters except 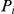 are assumed to be constant, substituting Eqs. (5) and (6) in Eq. (7) results in
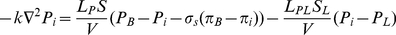
(9)Rearranging Eq. (9) for a spherical solid tumor with radius 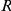,
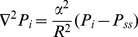
(10)



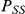
 is defined later by Eq. (14). Using the definition of Laplace operator, Eq. (11), in the spherical coordinate system, Eq. (10) is written as Eq. (12).
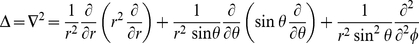
(11)

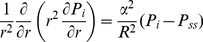
(12)in Eqs. (10) and (12), the ratio of interstitial resistance to vascular resistance is introduced in terms of 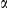, the dimensionless parameter defined by Eq. (13).
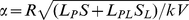
(13)

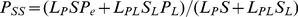
(14)the steady state pressure, 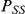, is the interstitial pressure at which the efflux from the vasculature and influx into the lymphatics are equal, and is defined by Eq. (14). Effective pressure, 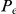, in Eq. (14), is a parameter defined by vascular pressure, plasma osmotic pressure, and interstitial osmotic pressure through Eq. (15).
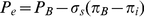
(15)


Applying the appropriate boundary conditions and also all the constants mentioned earlier, the governing equation, Eq. (10) or (12), can be used to calculate the interstitial fluid velocity (IFV) and interstitial fluid pressure (IFP) profiles in solid tumors. No lymph vessels in a solid tumor means 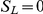; thus, Eqs. (12) and (13) can be simplified as follows:
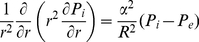
(16)

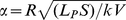
(17)in which the interstitial pressure that yields zero net volume flux out of the vasculature is called the effective pressure, 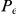, Eq. (15). The steady state pressure and effective pressure in solid tumors with no lymph vessels are the same. If 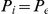, no exchange of fluid occurs between the interstitial space and blood vessels.

Due to symmetry, there is a no flux boundary condition at the center of the tumor; i.e.,
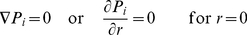
(18)


**Figure 3:**
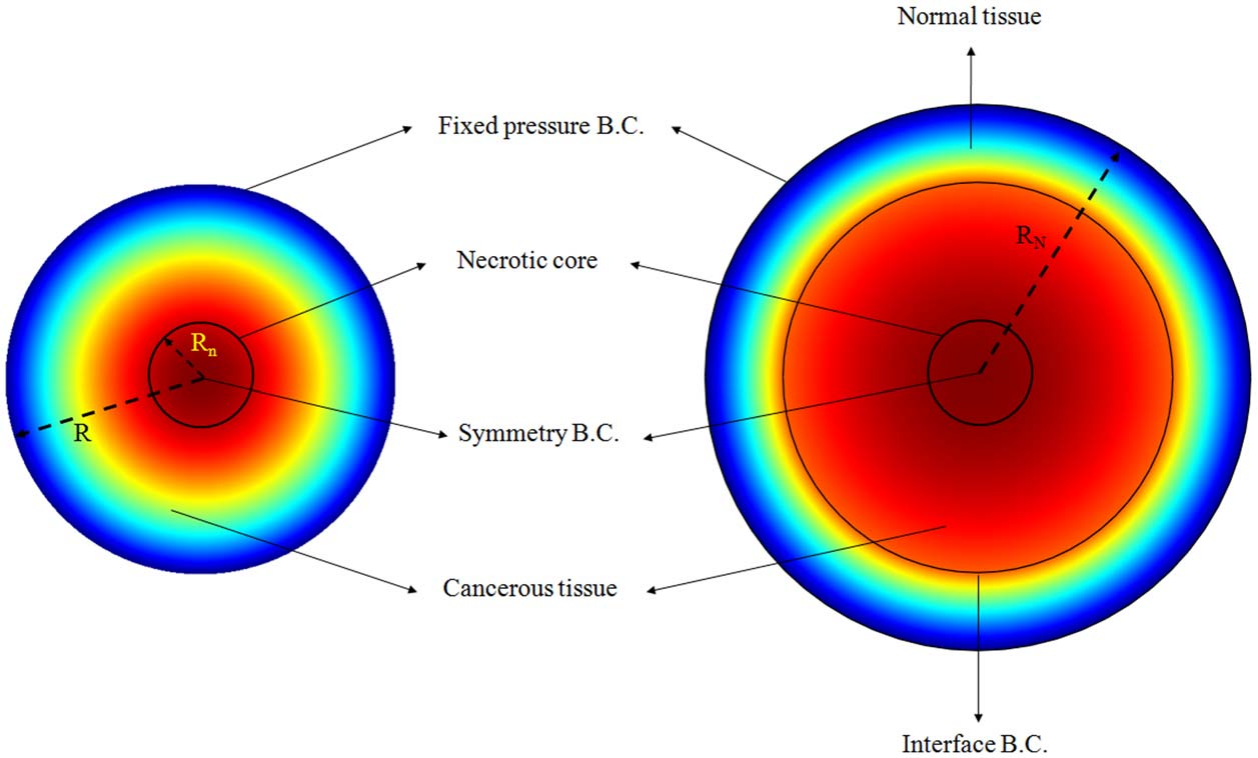
Two types of boundary conditions at the outer edge of the tissue.

At the outer edge of the solid tumor, 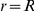, two types of boundary conditions are possible. In the first type, where the pressure in the surrounding tissue is fixed, the tumor pressure at the outer edge is the same as the surrounding pressure, 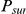.
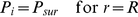
(19)This condition is applicable for an isolated tumor [39], [40]. In the second type, the solid tumor is surrounded by normal tissues. Pressure decreases smoothly over a distance; therefore, the continuity of pressure and velocity should be considered as an appropriate boundary condition for this case as the following conditions occur simultaneously:
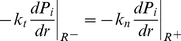
(20)

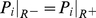
(21)where 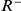 and 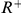 indicate the tumor and normal tissue radius at the outer edge of the solid tumor; 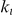 and 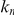 are the hydraulic conductivity of the interstitium in tumor and normal tissues, respectively. It should be noted that, in the second type, all the equations mentioned for the tumor tissue have to be solved for the normal tissue, as well. It is clear that for the normal tissue, far enough from the solid tumor that the pressure is constant, the first type of boundary condition, Eq. (19), must be applied. These two types of boundary conditions are shown in Fig. 3. The solution now can be obtained analytically or numerically to find the IFV and IFP profiles for each of the two boundary conditions. In this work, the numerical method has been used. An element based finite volume method (EB-FVM) is applied to discretize the equations. The EB-FVM has the capability of the finite element method (FEM) in handling complex geometries and also the sound physical-based properties of the finite volume method (FVM) [41]. The discretized form of the governing equations, in their general form, is then linearized and solved implicitly. The SIMPLE (Semi Implicit Method for Pressure Linked Equations) algorithm is used as the coupling method for pressure and velocity terms. Finally, the converged form of the solution is calculated using an iterative method. In order to improve the convergence rate, the method of successive over-relaxation (SOR) is applied with an under relaxation factor equal to 0.75. The criterion for the convergence is to reduce the residual by 6 orders of magnitudes. In order to check the grid independency of the code, the results for three different grids are compared, indicating the conservative property of the numerical method. Final choice of the grid includes 11904 control volumes.

The material properties for tumor and normal tissue were taken from the simulation studies of Jain and Baxter [13] and are shown in Table 1. It should be noted that tissue properties vary greatly among different organs for both normal and cancerous tissues; therefore, parameters introduced in Table 1 should be updated for new applications. As mentioned earlier, tissue transport properties are often anisotropic. Geometric and physiological properties of anisotropic and heterogeneous tissues affect drug delivery. This issue can be solved with the help of diffusion tensor imaging (DTI). A good application of this method in brain tumors is discussed by Linninger et al. [42].

**Table 1:** Material properties used in numerical simulations, as taken from [13].

Parameter	Tissue	Baseline value	Reference
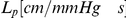	Normal	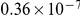	Rippe et al. (1978)
	Tumor	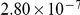	Jain (1987a)
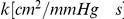	Normal	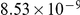	Swabb et al. (1974)
	Tumor	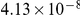	Jain (1987a)
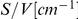	Normal	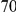	Pappenheimer et al. (1951)
	Tumor	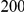	Hilmas and Gilette (1974)
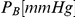	Both	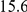	Brace and Guyton (1977)
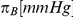	Both	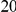	Brace and Guyton (1977)
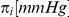	Normal	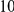	Wiederhielm (1979)
	Tumor	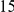	Jain (1987a)
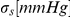	Normal	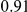	Ballard and perl (1978)
	Tumor	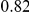	Curry (1984)

## Results

**Figure 4:**
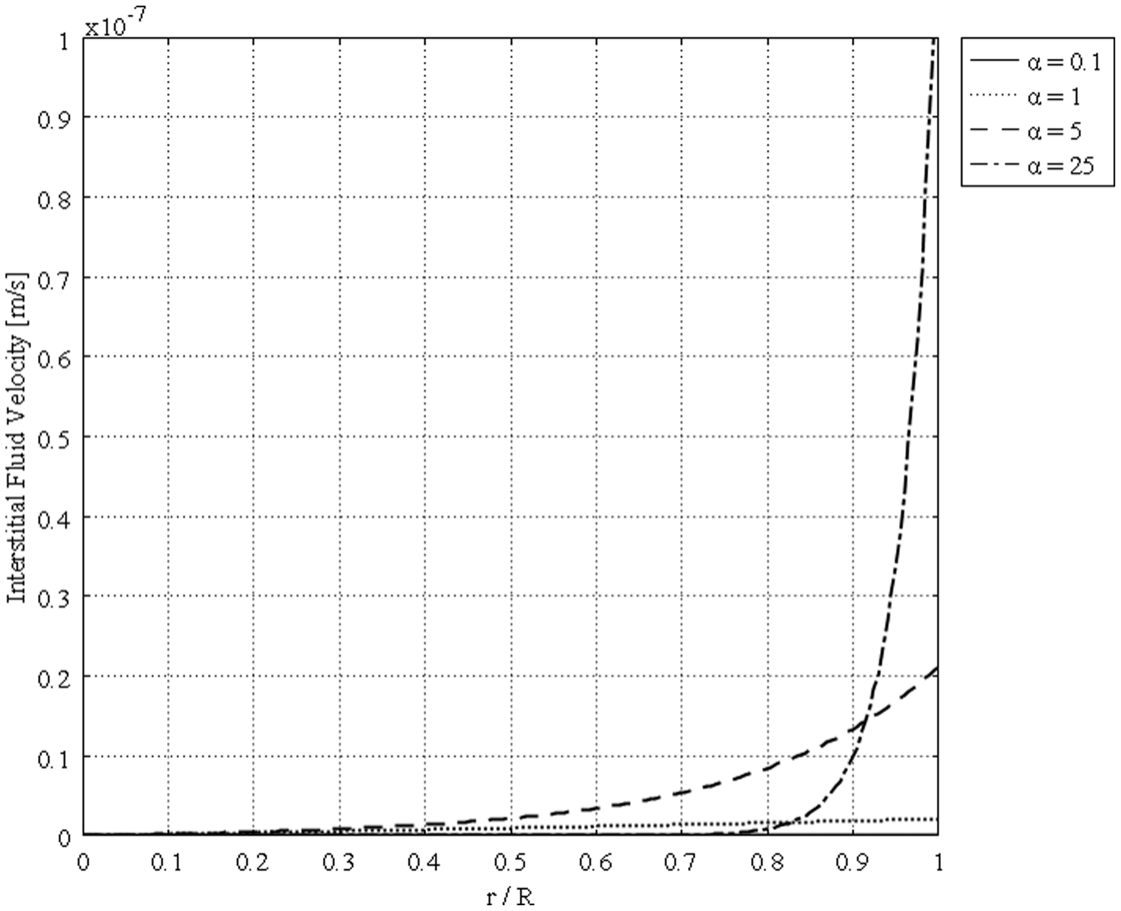
Interstitial velocity distribution in a 1 cm radius tumor, different values of 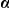, Eq. (16).

**Figure 5:**
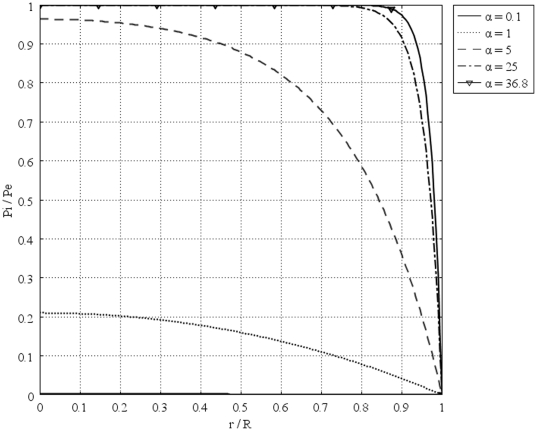
Dimensionless interstitial pressure distribution in the same tumor, different values of 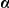.

**Figure 6:**
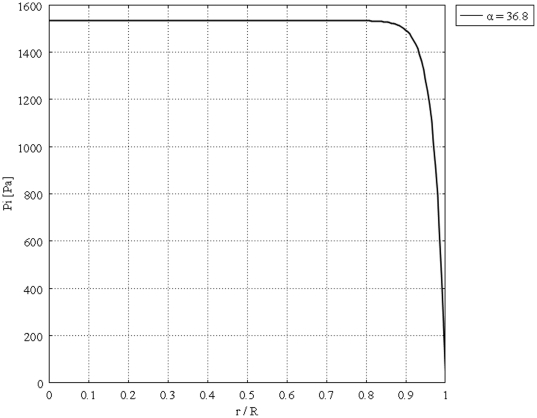
Interstitial pressure distribution in the same tumor (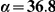).

**Figure 7:**
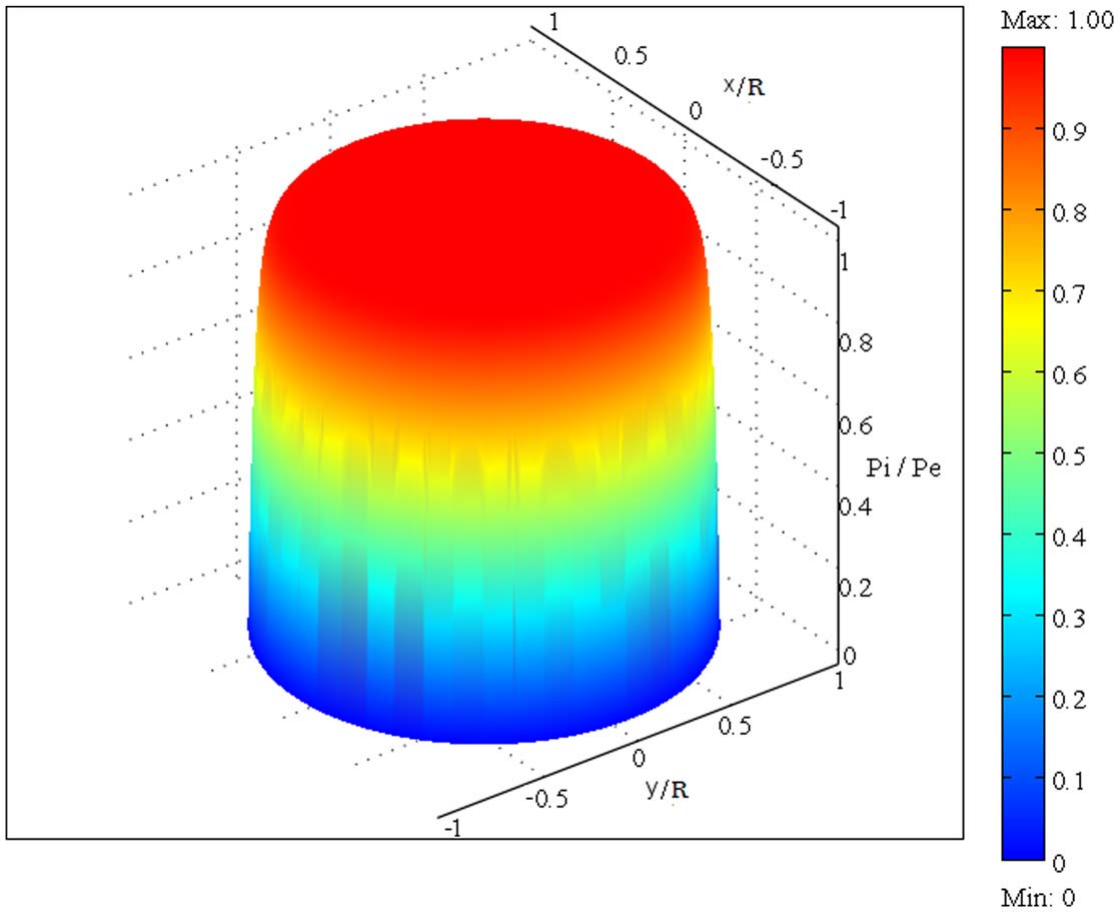
Three dimensional plot of Fig. 6, dimensionless interstitial pressure distribution, in the same tumor (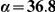).

**Figure 8:**
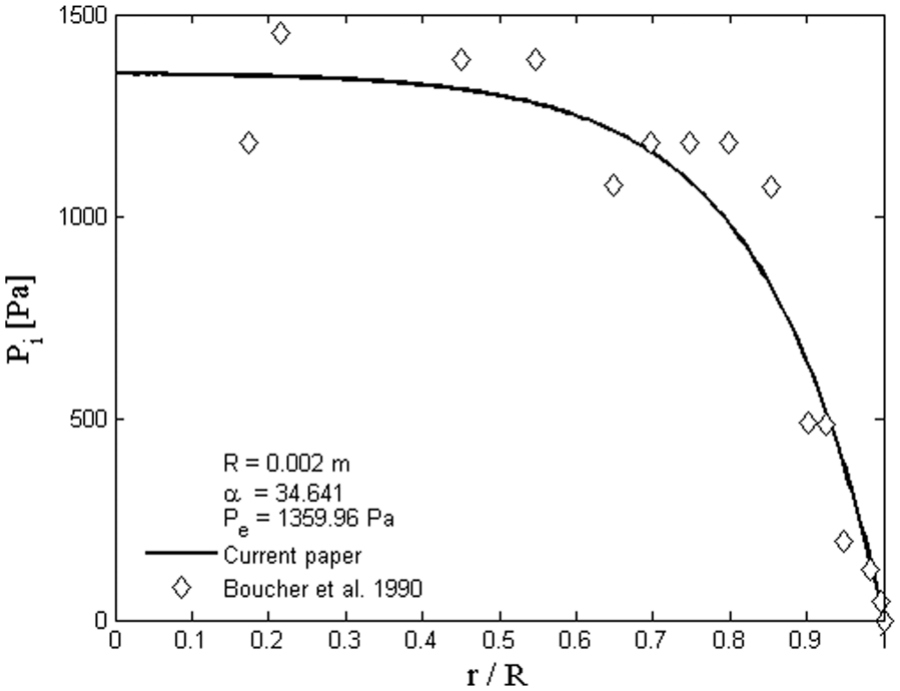
Comparison of the current paper with experimental data (mammary adenocarcinoma s.c.) by Boucher et al. [**15**].

**Figure 9:**
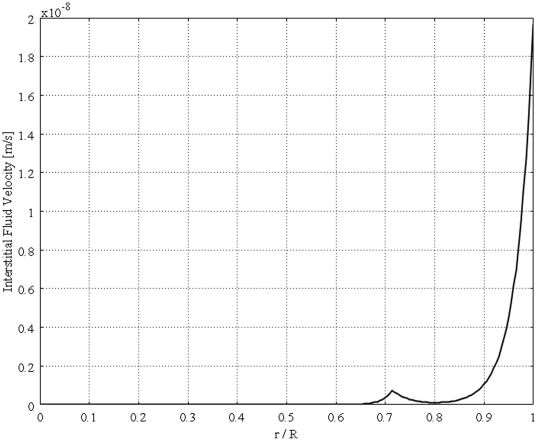
Interstitial velocity distribution in a 1.4 cm radius tumor and normal tissue.

**Figure 10:**
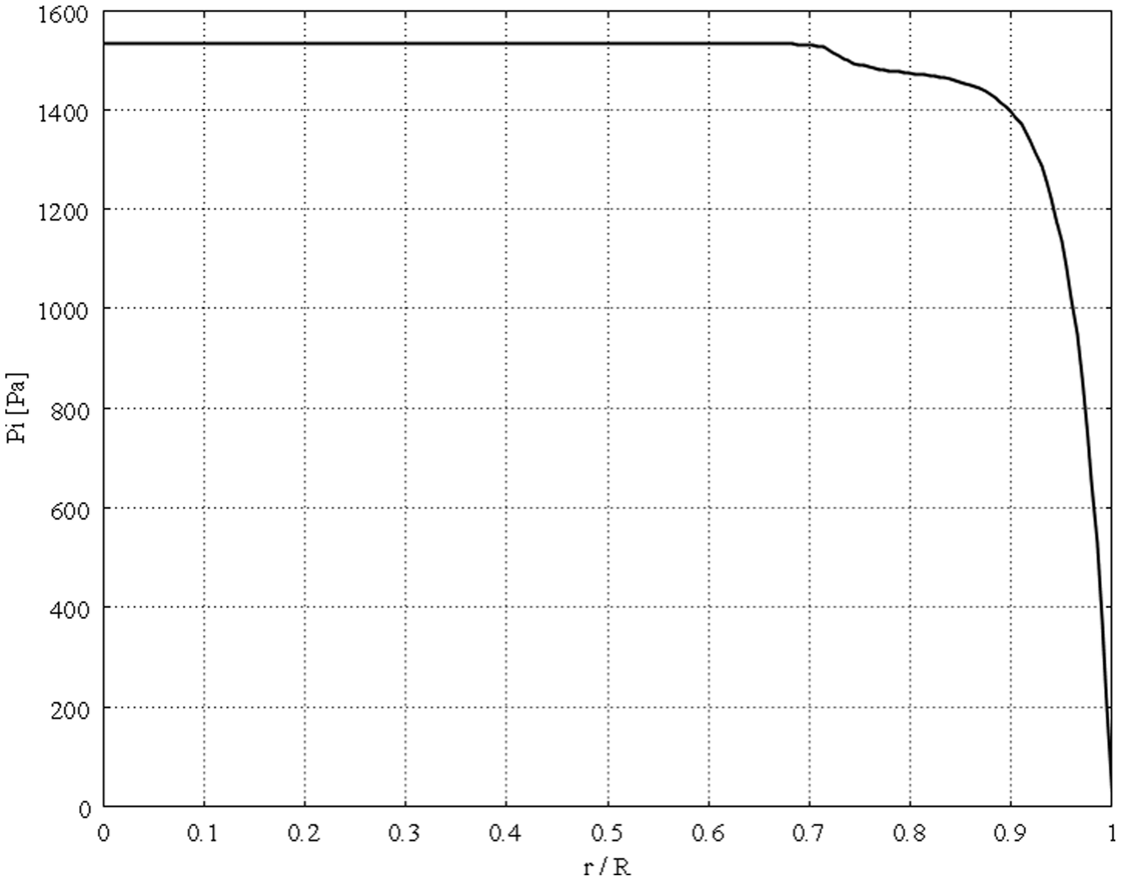
Interstitial pressure distribution in a 1.4 cm radius tumor and normal tissue.

Figure 4 shows the unidirectional interstitial fluid velocity distribution in an isolated solid tumor as a function of the dimensionless radius. This figure shows that the higher the value of 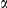 is, the steeper the velocity profiles will be. Figure 5 shows the unidirectional interstitial fluid pressure distribution in an isolated solid tumor as a function of the dimensionless radius. Low values of 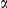 corresponding to flat curves show less resistance to fluid source, but high values of 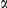 corresponding to sharp curves in the periphery of the tumor show greater resistance to fluid source, based on the definition of dimensionless parameter 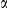. Figure 6 shows IFP distribution based on the parameters' values introduced in Table 1 which results in 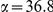. Experiments done by Baxter et al. were based on this value of 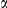 [3]. Three dimensional plot of Fig. 6, dimensionless interstitial pressure distribution, is shown in Fig. 7. All of these results agree well with experimental data [3], [4], [11]. Figure 8 shows the comparison of the current paper with experimental data (mammary adenocarcinoma s.c.) by Boucher et al. [15]. In this figure there is good agreement between model and experiment. Figures 9 and 10 show the IFV and IFP distribution for a solid tumor embedded in normal tissue as a function of the dimensionless radius, respectively. As mentioned earlier, boundary conditions for this case are different from those of an isolated tumor and are stated in equations (20) and (21).

**Figure 11:**
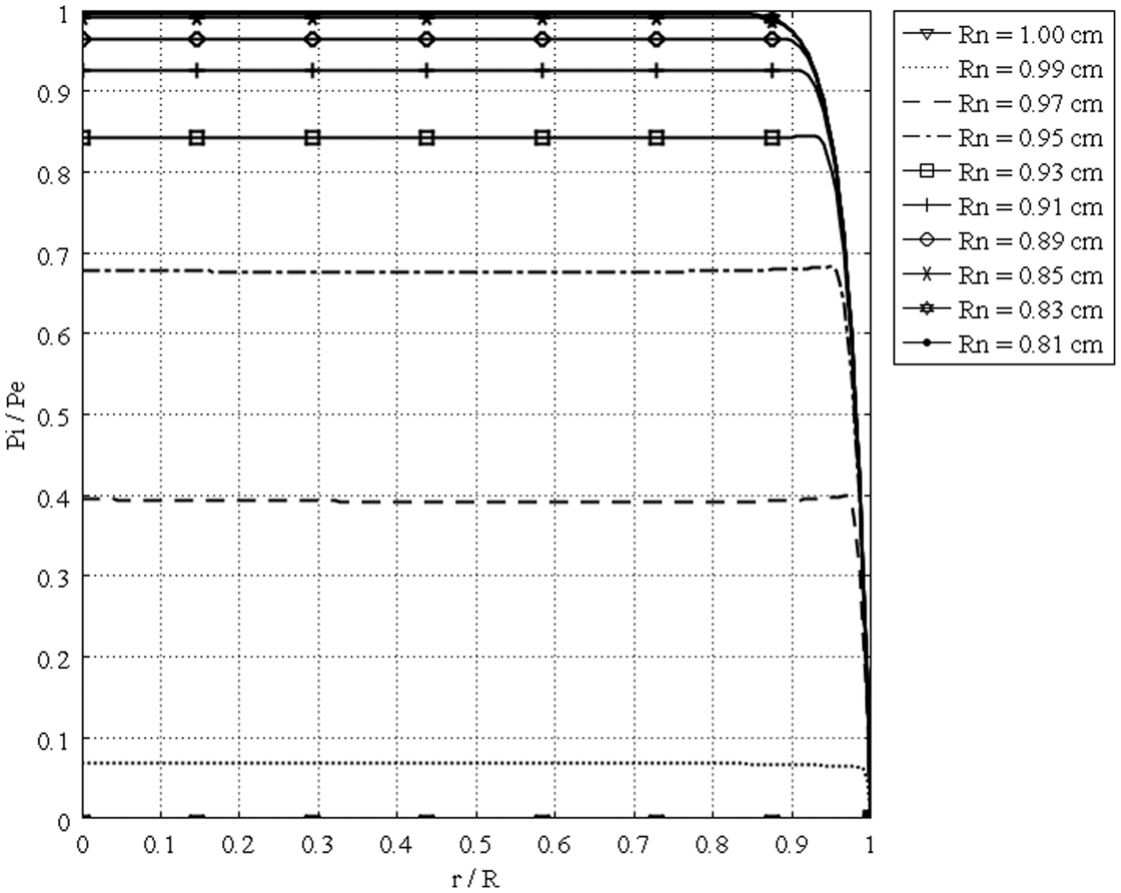
Interstitial pressure distribution in a 1 cm radius tumor, as a function of the dimensionless radius for different necrotic radii.

**Figure 12:**
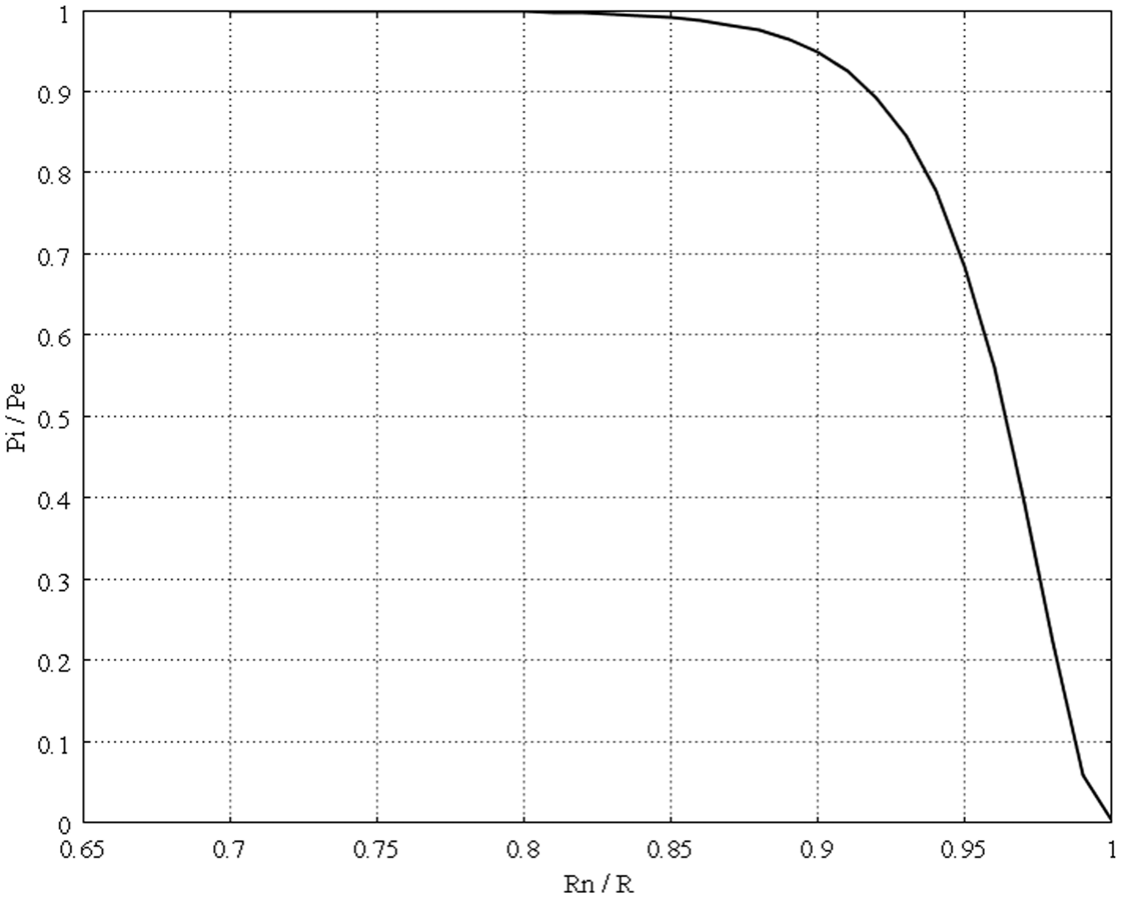
Interstitial pressure distribution at the center of a 1 cm radius tumor, as a function of the dimensionless necrotic radius.

**Figure 13:**
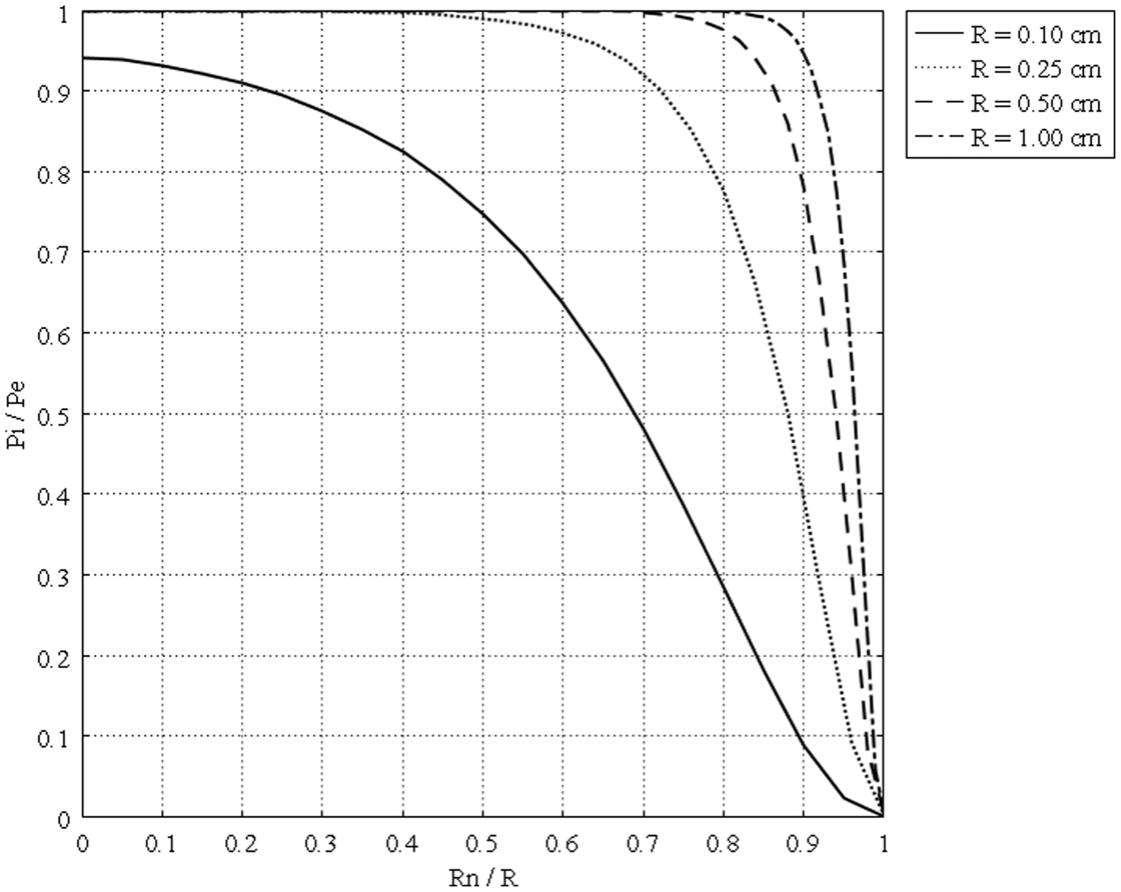
Interstitial pressure distribution at the center of different tumors, as a function of the dimensionless necrotic radius.

**Figure 14:**
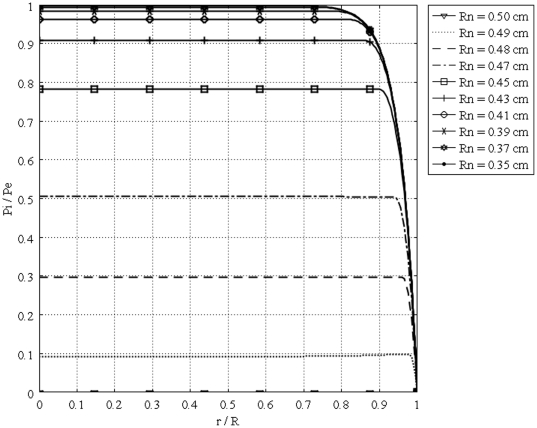
Interstitial pressure distribution in a 0.5 cm radius tumor, as a function of the dimensionless radius for different necrotic radii.

**Figure 15:**
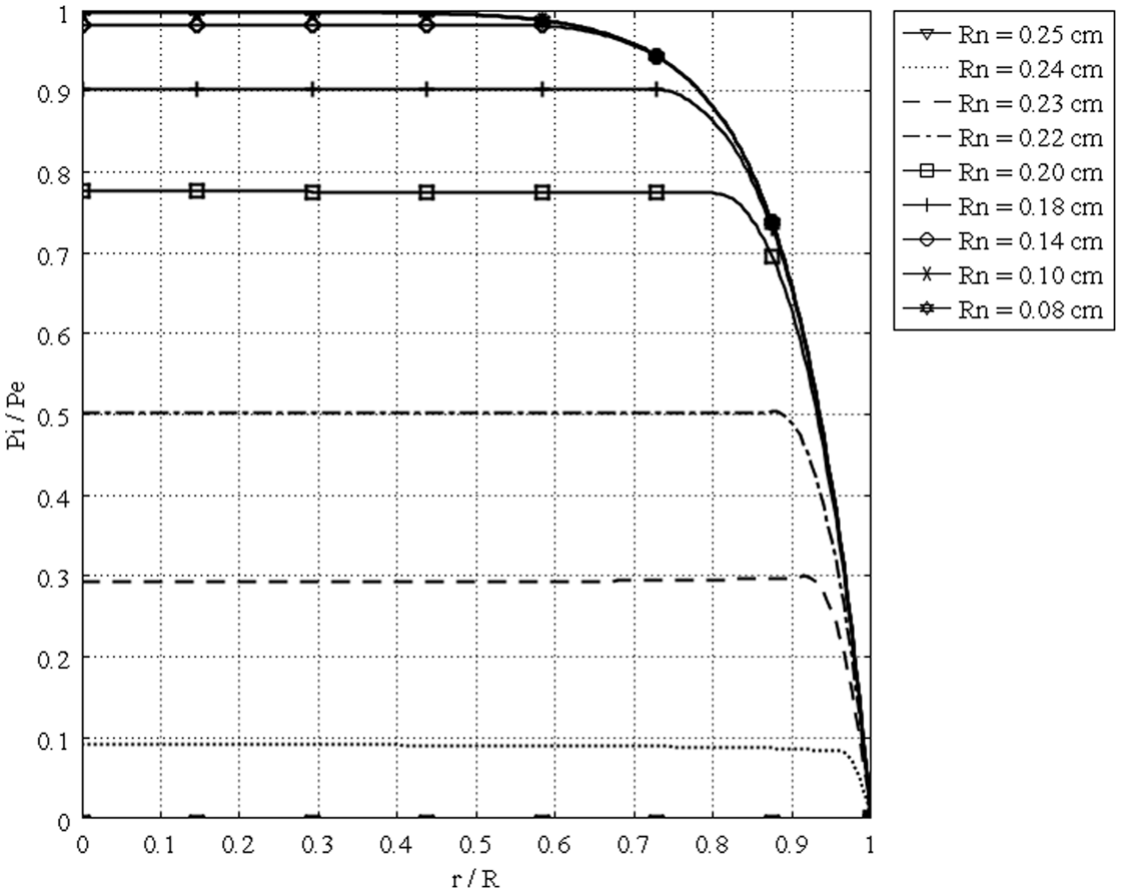
Interstitial pressure distribution in a 0.25 cm radius tumor, as a function of the dimensionless radius for different necrotic radii.

**Figure 16:**
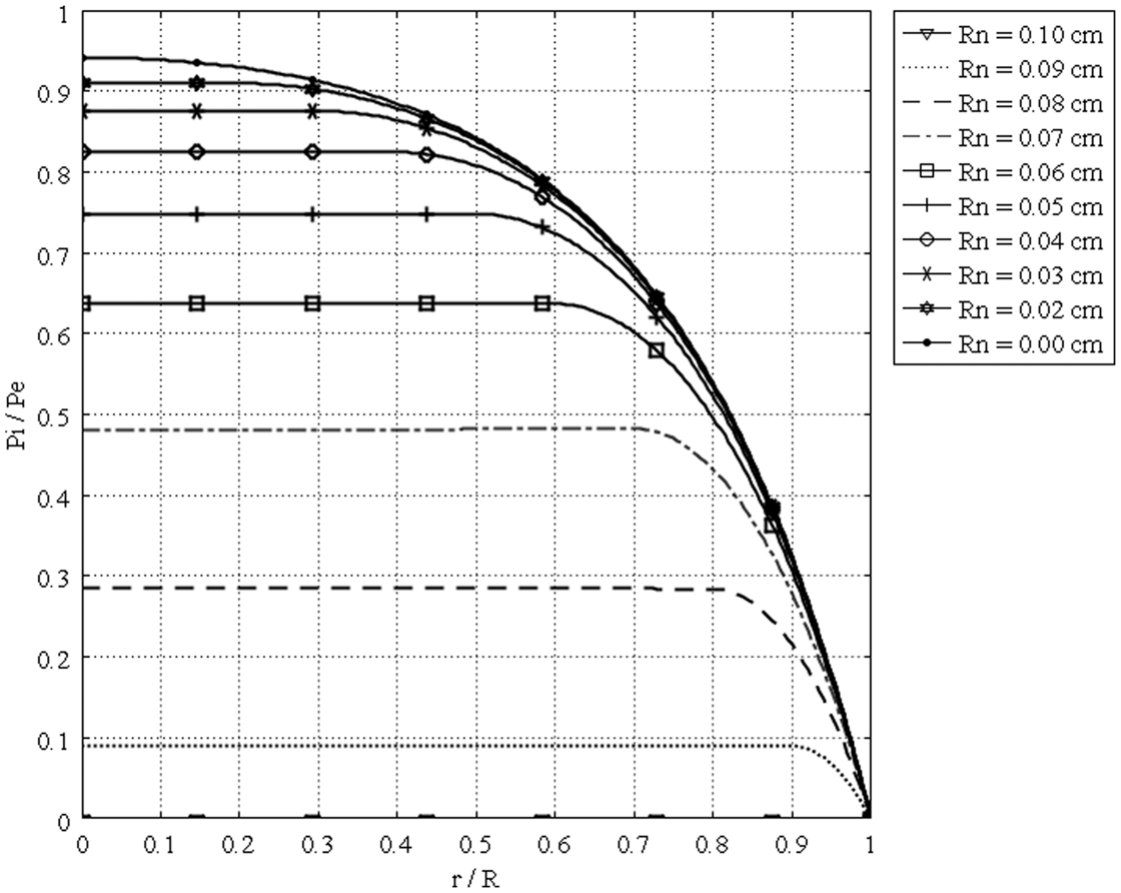
Interstitial pressure distribution in a 0.1 cm radius tumor, as a function of the dimensionless radius for different necrotic radii.

Figure 11 shows the interstitial pressure distribution in a 1 cm radius tumor, as a function of a dimensionless radius for different values of necrotic radii. This figure shows that an increase in the necrotic radius decreases the maximum pressure inside the tumor, and obviously, when the entire tumor is necrotic, with no vasculature, the IFP is zero. On the other hand, for a necrotic radius below a certain size, IFP has its maximum value, which is the effective pressure, 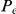, and this limited size, which can be considered as a critical necrotic radius or 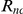, can be interpolated from a graph such as Fig. 12. The same graph for all sizes of solid tumors studied in this paper is also shown in Fig. 13. From this figure, for instance, for tumors below a certain size (in this case 0.1 cm), reaching effective pressure, even with a zero value for their necrotic radius, is not possible. Figures 14, 15 and 16 show the same parameters as Fig. 11 for the other three tumor sizes, which follow the same behavior as explained for Fig. 11.

## Discussion

The calculated value of IFV for the periphery of an isolated tumor, shown in Fig. 4, is on the order of 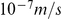, a finding that agrees well with experimental data in the literature [3], [4], [11], [40]. These results predict that the fluid filtration is negligible throughout most part of the tumor and occurs mostly from vessels in the periphery, well vascularized region. Interstitial fluid pressure distribution for different values of 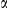 and different boundary conditions, Figs. 5 and 10, shows that IFP is elevated throughout the tumor and goes down sharply in the periphery of an isolated tumor or at the normal tissue around an embedded solid tumor. The immediate result of this high IFP is decreasing blood flow and therefore insufficient delivery of drug. This general trend for IFP leads to low filtration of drug from blood vessels in the center of the tumor and high filtration of drug in the tumor periphery, as shown in Fig. 1. On the other hand, the large pressure gradient results in an outward convective flow that washes out the drug extravasated from blood vessels at the tumor periphery. All of these phenomena are indicated in Fig. 1, schematically. In both cases, embedded and isolated tumor, IFP approaches 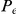, effective pressure, in the center of the tumor where the fluid source is minimal, as shown in Figs. 5 and 10. On the other hand, in the periphery, the opposite scenario occurs; there is minimum pressure and maximum fluid source. Based on the results of this study, drug delivery can be enhanced by decreasing IFP in the center of the tumor. This may not be easy to do but there are some physical and enzymatic methods which can be applied. For instance, blocking the integrin links between interstitial matrix and cells decreases IFP and enhances tissue fluid content. Also, irradiation by remodeling extracellular matrix decreases IFP in solid tumors [43]. Comparing IFP distribution in tumors with different radii shows that IFP increases with tumor size. This study introduces two new parameters, the critical tumor radius and critical necrotic radius. For tumors below the critical tumor radius, the maximum interstitial fluid pressure is less than effective pressure, no matter what the value of the necrotic radius is. In fact, the transport of the drug to the center of smaller tumors is much easier than transport to the center of a tumor whose radius is greater than the critical tumor radius, as the maximum IFP is much lower than effective pressure, 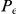. This study also shows that there is a critical necrotic radius, below which the interstitial fluid pressure at the center of the tumor is at its maximum value. If the tumor radius is greater than the critical tumor radius, this maximum pressure is equal to the effective pressure.

### Conclusions

Numerical solutions for the simplest case of a homogeneous and alymphatic tumor demonstrate that, in a uniformly perfused tumor, high interstitial pressure is the main cause of heterogeneous drug distribution. The main assumption used to reach this conclusion is that drug particles flow with the interstitial fluid. The distribution of interstitial fluid pressure and velocity have been calculated by numerical solutions to the governing equations. Comparison of these numerical solutions and experimental data in the literature shows that the maximum value for the interstitial pressure occurs at the center of the tumor and decreases towards the periphery and that the numerical values of interstitial fluid velocity and the experimental results reported in the literature agree. Interstitial fluid pressure is not uniform whether the tumor vasculature is homogeneous or heterogeneous. Thus, in addition to the heterogeneous distribution of blood supply, high interstitial pressure plays a significant role in drug distribution in a solid tumor.

This study also shows that an increase in the necrotic radius decreases the maximum pressure inside the tumor; the tumor that is completely necrotic has no vasculature, and thus its interstitial fluid pressure is zero. This study introduces two new parameters, the critical tumor radius and critical necrotic radius. Simulation results show that for tumors below the critical tumor radius, the maximum interstitial fluid pressure is less than effective pressure (a parameter determined by vascular pressure, plasma osmotic pressure, and interstitial osmotic pressure); therefore, the transport of the drug to the center of smaller tumors is much easier than transport to the center of a tumor whose radius is greater than the critical tumor radius, as the maximum interstitial fluid pressure is much lower than effective pressure. This study shows that there is a critical necrotic radius, below which the interstitial fluid pressure at the center of the tumor is at its maximum value. If the tumor radius is greater than the critical tumor radius, this maximum pressure is equal to the effective pressure. At above this critical necrotic radius, the interstitial fluid pressure at the center of the tumor is below effective pressure.

The numerical model investigated here can be further extended to apply to anisotropic tissues in terms of properties and geometry. Capillary distribution in real tissues is heterogeneous and non-uniform, this numerical model can handle this issue as well. Microcirculation studies show that for some drugs the relative size of nanoparticles is comparable to the capillary diameter; therefore, flow field has to be modeled as a two phase flow. Numerical method introduced in this paper has the capability of doing such a two phase flow model.
